# Two-Step Matching Approach to Obtain More Control Points for SIFT-like Very-High-Resolution SAR Image Registration

**DOI:** 10.3390/s23073739

**Published:** 2023-04-04

**Authors:** Yang Deng, Yunkai Deng

**Affiliations:** 1 Space Microwave Remote Sensing System Department, Aerospace Information Research Institute, Chinese Academy of Sciences, Beijing 100190, China; 2 School of Electronic, Electrical and Communication Engineering, University of Chinese Academy of Sciences, Beijing 100039, China

**Keywords:** VHR SAR image, SITF-OCT, CPs

## Abstract

Airborne VHR SAR image registration is a challenging task. The number of CPs is a key factor for complex CP-based image registration. This paper presents a two-step matching approach to obtain more CPs for VHR SAR image registration. In the past decade, SIFT and other modifications have been widely used for remote sensing image registration. By incorporating feature point location affine transformation, a two-step matching scheme, which includes global and local matching, is proposed to allow for the determination of a much larger number of CPs. The proposed approach was validated by 0.5 m resolution C-band airborne SAR data acquired in Sichuan after the 2008 Wenchuan earthquake via a SAR system designed by the IECAS. With the proposed matching scheme, even the original SIFT, which is widely known to be unsuitable for SAR images, can achieve a much larger number of high-quality CPs than the one-step SIFT–OCT, which is tailored for SAR images. Compared with the classic one-step matching approach using both the SIFT and SITF–OCT algorithms, the proposed approach can obtain a larger number of CPs with improved precision.

## 1. Introduction

Synthetic-aperture radar (SAR) plays a decisive role in the context of Earth observation technologies due to its all-weather capability. Different SAR imaging systems, extending over many SAR image applications (such as overseas oil leak detection; marine monitoring, such as [1,2] earthquake and landslide disaster monitoring and assessment [3,4]; reservoir and bridge infrastructure deformation monitoring [5,6]; urban building deformation monitoring [7,8]; the fine-structure imaging of buildings [9]; wisdom city applications), are based on high-precision SAR image registration technology. Image registration refers to the different imaging conditions of two or more remote sensing images through analysis and processing to provide accurate geographical location information, the maximum degree of reduction in the target object information, and more accurate analysis, as well as to extract the depth of the image information, such as in the Yangtze River water extraction area [10,11] and along railway settlements [12,13], for feature classification and crop recognition [14], landslide change detection [15,16], accurate drone positioning [17,18], etc. Compared with space-borne low-resolution SAR image registration, airborne very-high-resolution (VHR) SAR image registration is much more difficult for the following reasons: (1) the flight path and attitude of the airborne platform are unstable, which causes different imaging geometries for two flights; (2) the high-resolution character provokes an obvious slight ground change in two image pairs; (3) it is difficult to entirely correct the antenna pattern weight, which could cause the image intensity to differ in various range units; (4) complex topography suffers from the different distortions of two VHR SAR images. The most common method for image registration is the use of control points (CPs) to estimate the geometry distortion, followed by the mapping of one image onto the other’s coordinate system. For simple distortion, only a few CPs are sufficient to estimate the distortion model. However, for complex distortion, a more complex model is needed, which requires more CPs. Thus, the number of high-quality CPs is key for this challenging task.

There are many features in SAR images, such as point, line, and surface features, and many classic feature extraction algorithms have been proposed and applied to remote sensing image registration. In the 1980s, Moravec proposed the concept of angular point detection [19]. The algorithm detects angular points by detecting the average change in the window moving in different directions. The disadvantages of the detection operator are that it is easily affected by edge points and noise and it cannot deal with cases of rotation. Later, Harris and Stephens proposed the Harris operator. The angular point detection operator is simple to calculate and can deal with the rotation and gray changes, but it can only obtain pixel-level feature points [20]. On this basis, Lowe proposed a better-performing feature transformation in 1999: SIFT [21], and then summarized and refined the SIFT feature descriptors to have grayscale, rotation, and affine invariance [22]. Bay et al. proposed the SURF algorithm, and they refined it in 2008 [23]. Z. Zhou introduced the Hu invariant moment into the grayscale-matching algorithm [24]. Z. Yang used the modified SIFT features for SAR image matching [25]. Z. Xiong matched the optimized SURF features using the RANSAC method and applied them in the SAR/INS combined navigation system [26]. Du Jiang used two SURF matching-point-intersection and RANSAC methods for mismatch elimination [27]. In 2019, R. Luo used the significance-weighted method to reduce the number of SURF feature points to achieve aerial image matching [28]. According to the two disadvantages of the correlation coefficient method, Y. Wang adopted fast Fourier transform and integrated imaging on the basis of block division, which achieves more robust and faster remote sensing image registration [29] compared with normalized mutual information. Pallotta et al. introduced the subpixel registration of remote sensing images by performing parabolic interpolation around the peak of reciprocal relations [30].

As a classical algorithm, the scale-invariant feature transform (SIFT) is applied to match local features by a feature detector and feature descriptor. SIFT is adapted to remote sensing image matching, as it is invariant to image scaling and rotation and partially invariant to changes in illumination and the look angle. A number of variants of the SIFT algorithm have been developed for SAR image registration, such as SIFT-octave (SIFT-OCT) [31], bilateral filter SIFT [32], adapted anisotropic Gaussian (AAG) SIFT [33], SAR-SIFT [34], and the uniform SIFT-like algorithm [35]. An enhanced version of SIFT termed principal component analysis scale-invariant feature transform (PCA-SIFT) can provide more distinctive representation for local image descriptors, which obtain superior matching results [36]. Furthermore, another variant, affine-SIFT, is a fully affine-invariant feature detector that can handle large affine distortions [37]. SIFT-based methods have been successfully used in remote sensing data due to their excellent performances [38]. Although many modifications of the SIFT algorithm have been proposed in the literature [36,39,40,41], SIFT and its variants are suitable for the registration of optical images with relatively weak noise, but not for SAR images with multiplicative noise. To adapt the algorithm to SAR images and reduce the effect of speckle noise, Wang et al. [32] suggest the use of anisotropic scale space to replace the Gaussian one. Hence, the first octave is ignored, and the image is filtered using an infinite-size symmetrical exponential filter for reducing the speckle noise effect; however, it blurs the details in the image [42]. The AAG filter preserves the edges better than the Gaussian filter and is more robust to noise than the nonlinear diffusion formulation [43]. Some methods, such as dual matching and random sample consensus (RANSAC), are used to filter false-match pairs [44].

Despite having achieved some good performances, these algorithms fail to take the statistical specificities of SAR images into account. To address this problem, Dellinger et al. presented a SIFT-like algorithm dedicated to SAR imaging (SAR-SIFT), which relies on the new gradient by the ratio (GR) method and the new multiscale SAR-Harris space [34]. The SAR-SIFT features have been applied in many SAR image registration algorithms [45,46]. Liu et al. proposed a method combining SIFT and the block-matching method to overcome the drawbacks of these algorithms separately [47]. Yu et al. proposed a two-step algorithm where coarse registration is performed using affine SIFT, and the radial basis function is used to model the local deformation [48]. Zhou et al. enhanced the keypoint descriptor by using improved dominant orientation assignment and support regions to improve the registration results [49]. Paul et al. [46] proposed the I-SAR-SIFT algorithm based on SAR-SIFT and UR-SIFT [39], which greatly improves the influence of speckle noise on the features and the matching performance of the algorithm based on the local-matching strategy based on Delaunay triangulation. According to Divya et al. [50], different images have different geometry and intensity changes based on the structure tensor of the SIFT algorithm (ST-SIFT, the algorithm using the structure tensor filter image), and even after many iterations of filtering, it can still better retain the image edge details and corner information. Multifeature extraction is performed using SAR-SIFT (for corner features) and R-SIFT (for texture features) to obtain more feature points in [51]. A nonlinear diffusion method is used to construct the scale space and ROEWA to find the gradients in [52], and the method uses phase congruency [53] to eliminate the outliers. The spatial correlation strategy based on stationary wavelet transform is utilized in [54] to select reliable keypoints from the SIFT algorithm to reduce the influence of speckle noise. In [55], an improved anisotropic Gaussian scale-space SIFT is used to find the keypoints for SAR image registration.

Most of these methods attempt to find better feature representation to obtain a larger number of CPs. However, our work uses a different perspective. Instead of improving the feature representation algorithm, this paper introduces a CP-matching scheme that can also obtain a larger number of accurate CPs. The proposed approach uses a two-step matching strategy under the SIFT-like SAR image registration scheme. The proposed two-step matching approach was evaluated on both the SIFT and SIFT-OCT algorithms. Even the original SIFT algorithm, which is not suitable for SAR, can achieve a much better result than one-step SIFT-OCT, which is tailored for SAR imaging. Compared with the classic one-step matching, our experiments show that two-step matching can obtain more accurately matched CPs, and it also improves the matching accuracy.

In the following section, the motivation and proposed two-step matching scheme of this paper is introduced. The evaluation dataset and experiment are presented in Section 3. In Section 4, a brief conclusion is presented.

## 2. Two-Step Matching

### 2.1. Registration Model

Let $I_{m}$ and $I_{s}$ be master and slave images, respectively, covering the same area but with different imaging geometries. Given a ground point, we have two different image coordinate pairs: ($x_{m}$, $y_{m}$) and ($x_{s}$, $y_{s}$). The relationship between the two coordinate pairs can be described as follows:

(1) $$\left(x_{m},y_{m})=T(\alpha ,x_{s},y_{s}\right)$$

where T(•) is a transformation function, which is used to model the image distortion, and α is a parameter vector of T(•). The task of registration is to find an appropriate model (T) and the corresponding optimal parameter estimation of vectors (α) so that the slave image can be resampled onto the master image coordinate system.

First, the model function (T) is defined based on a specific task, and then CPs are used to estimate the parameters. There are two major function classes for modeling distortion in SAR images according to the literature available. The first is global transformations, such as a similarity transformation, affine transformation, projective transformation, and polynomial transformation. These global transformations only require the estimation of a few parameters; therefore, relatively fewer high-quality CPs (more CPs lead to more stable estimations) are needed. Global transformations can only model relatively simple distortions, which do not always satisfy the requirement of VHR SAR image distortions. The second function class is local transformations, such as thin-plate spline (TPS) [56] and triangulated irregular network (TIN) [57], which model more complex distortions using local functions. For these local transformations, the number of CPs directly affects the registration performance. For VHR airborne SAR images with different imaging geometries and ground surface changes, the distortion of the image varies with the topography, and these local transformations are more suitable in these circumstances. The number of CPs should be large enough to obtain an accurate registration result.

### 2.2. Motivation for Two Steps

The traditional registration process based on control points usually contains three steps: the automatic extraction of control points, the estimation of the mapping function, and the interpolation of the auxiliary image resampling. Because the method of interpolating the auxiliary image to the main image reference coordinate system is optimal [58], this study focuses on the automatic extraction of control points and the modeling of the distortion mapping function. The principle and specific steps of the SIFT algorithm can be referred to in [22], where it is assumed that the feature points were extracted through this algorithm. Let $v_{}$ be the feature descriptor vector of a keypoint. The similarity of Point 1 and Point 2 is defined by the Euclidian distances of $v_{1}$ and $v_{2}$:

(2) $$d={\left\|v_{1}-v_{2}\right\|}_{2}$$

This means that the lower value of d indicates higher similarity. The first (1st) and second (2nd) similar feature descriptor vectors of the $v_{}$ are defined by $v^{1}$ and $v^{2}$, respectively, which correspond to the lowest and second lowest Euclidian distances to the $v_{}$, respectively. Supposing the norm of the feature descriptor vector is 1, which is ensured in the SIFT-like keypoint extractor, the contrast is defined as follows:

(3) $$c=\frac{\arccos{\left\|v^{1}-v\right\|}_{2}}{\arccos{\left\|v^{2}-v\right\|}_{2}}$$

where arccos represents the arccosine function. The lower value of c corresponds to higher contrast.

The traditional one-step matching calculates the d and c of every extracted fea- ture between the master and slave images. The feature points with the smallest d are found, and the c is assessed as to whether it is smaller than a predefined threshold (θ). If the feature points satisfy these requirements, then they are chosen as matched points.

**Figure 1:**
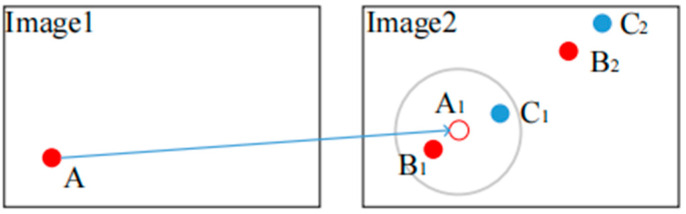
Description of two-step matching principle.

However, there are several defects of this one-step matching, which are illustrated in Figure 1. Assuming that we want to find a best match point of A in Image 1 from Image 2 (see Figure 1), we calculate the distance (d) between A and every keypoint in Image 2. Finding similar feature points in a larger SAR image is easier.

In the first case, supposing that the correct tie point of A_is_ $B_{1}$, when the acquisition time and imaging geometry of the two images differ, we may find the best match to be $B_{2}$. However, as the true tie point is $B_{1}$, the distance (d) of A and $B_{1}$ would be small. Suppose $B_{1}$ is selected as a 2nd similar point of A; the contrast (c) would be large. If $B_{1}$ is not selected as a 2nd similar point, then another point with a lower d would be selected, which also means large c. In both conditions, no point will be found as a matched point of A. However, this inference is not absolutely true; if the two images changed greatly, then the distance (d) of $B_{1}$ and A is large, and the incorrect match point ($B_{2}$) can be selected. These three conditions are undesirable.

In the second case, supposing that the best match is the correct tie point ($B_{1}$), the 2nd similar point is $C_{2}$. With the increase in the resolution and image scale, the probability of finding a 2nd similar point with a small d increases. In this condition, the contrast (c) may be very large. If the threshold value (θ) is small, then the point $B_{1}$ will not be selected. If the threshold (θ) is large, then $B_{1}$ will be selected, but so will many incorrect points, such as $B_{2}$ in the first case described above.

The proposed two-step matching scheme solves these problems. By using a global-matching and keypoint affine transformation, the location point (A) is projected onto the coordinate system of Image 2 (i.e., $A_{1}$). By defining the searching scope (the large gray circle), most of the 1st and 2nd interferences, such as $B_{2}$ and $C_{2}$, are eliminated. With a smaller searching scope, the probability of interference appears to decrease, the contrast (c) is decreased, and the searching speed is accelerated.

### 2.3. Two-Step CP Extraction Scheme

**Figure 2:**
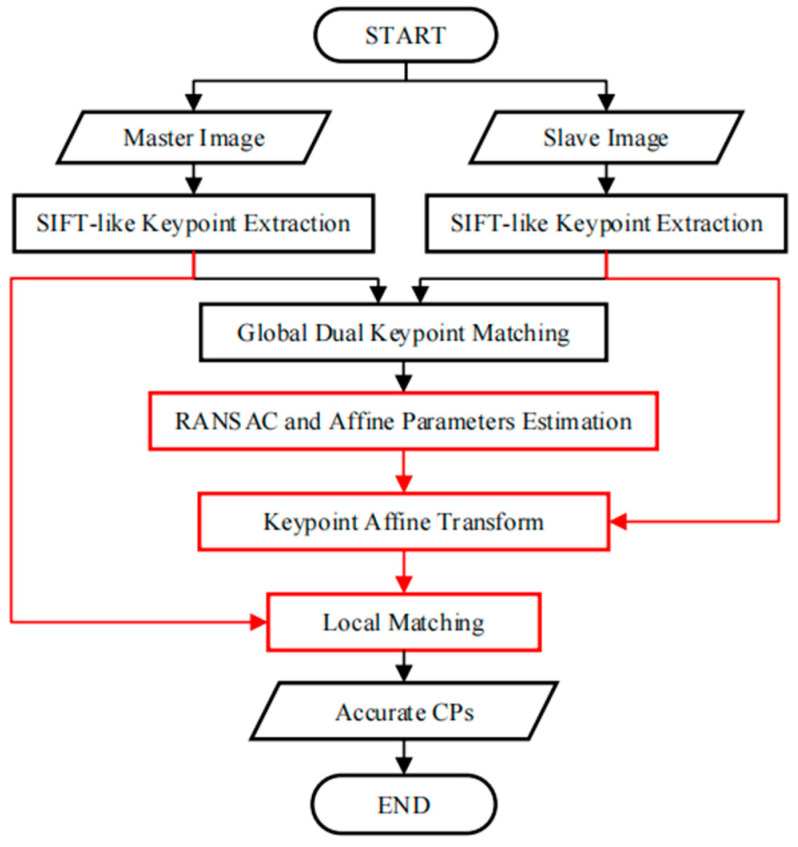
Procedure of two-step automatic CP extraction.

The scheme of the proposed automatic CP extraction approach for VHR SAR image registration is presented in Figure 2. The differences between our proposed method and the traditional one-step scheme are highlighted by the red-colored rectangles in Figure 2. The detailed algorithm is as follows:

(1) SIFT-like keypoint extraction. Extract keypoints and their descriptors from both the master and slave images using a SIFT-like algorithm (i.e., SIFT [22] or SIFT-OCT [31]);

(2) Global dual matching. For each keypoint of the master image, find its corresponding point in the slave image with the contrast (c) lower than the threshold (θ) as the forward match pair. For every keypoint of the slave image, find the backward match pair using the same method as for the forward match pair. Only the identical pairs in both the forward and backward matches are kept as global dual-matched point pairs;

(3) RANSAC and affine parameter estimation. Use affine transformation as the distortion model. Perform the RANSAC [44] method to remove false matches and obtain the affine transformation parameters ($a_{f}$);

(4) Keypoint location affine transformation. Apply the estimated affine transformation on every keypoint from the slave image. After this transformation, all the keypoints will be in the coordinate system of the master image. This transformation does not need to be accurate, but the displacement required is small. Moreover, the transformation is only applied to the keypoint of the slave image, excluding image resampling and interpolation, which is efficient;

(5) Local dual matching. Like global dual matching, local dual matching only searches for points within a small-radius (R) neighborhood. As the R is small, only a few keypoints are calculated, which results in higher efficiency and a reduction in interference points. The original locations of the local dual-matched points are expected to be the selected CPs.

## 3. Experimental Results and Analysis

### 3.1. Dataset

**Figure 3:**
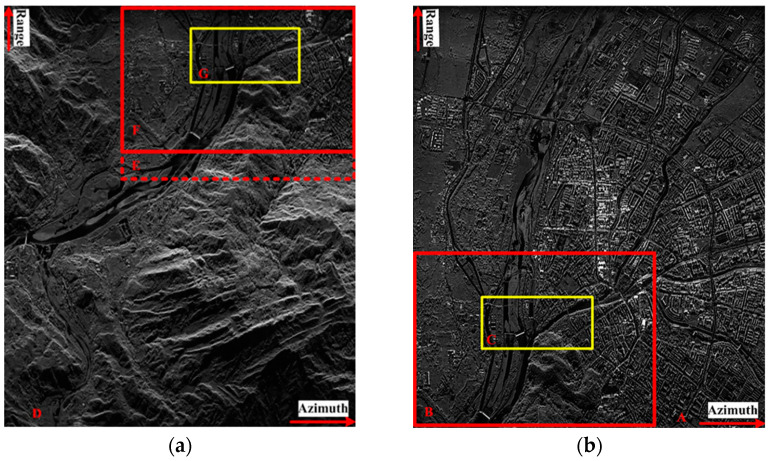
Airborne SAR images used in this paper: (**a**) master image; (**b**) slave image.

To show the improvement in the proposed method, a challenging airborne VHR SAR image registration task was performed. Images from a C-band airborne SAR sensor, which was designed by the Institute of Electronics, the Chinese Academy of Science (IECAS), were used in this work. Both images were acquired on 27 May 2008, for Wenchuan earthquake rescue purposes. The resolutions of both the slant range and azimuth direction are 0.5 m. As the two images are from different flights, the covered area in Figure 3a is located at the near range, while that in Figure 3b is at the far range. Thus, they have different ground-range resolutions. We labeled the images with characters from ‘A’–‘G’: ‘A’ and ‘D’ refer to the full-scene master and slave images, respectively, and ‘B’ and ‘F’ are the overlapping areas of both images, respectively. The sizes of ‘B’ and ‘F’ are not the same because these two images were acquired with different imaging geometries. ‘E’ has the same image size as ‘B’, but it is considerably larger than ‘F’. The intensities of the different subareas of the overlapping images are not identical because the antenna pattern weight was not entirely corrected. To facilitate discussion and evaluation, we manually selected subimages ‘C’ and ‘G’, which are located in nearly the same location and have complex land covers, including buildings, trees, rivers, and mountains. The existence of complex topography in VHR SAR images makes registration more difficult, which makes this task a typical example for analysis.

### 3.2. Analysis

**Figure 4:**
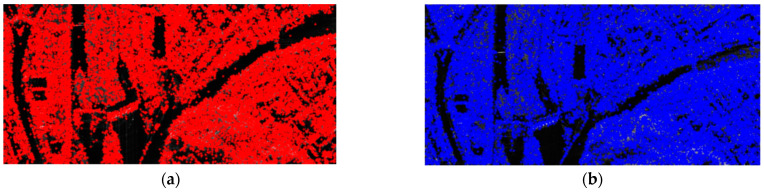
Keypoints extracted from SIFT: (**a**) keypoints of master image; (**b**) keypoints of slave image.

Both SIFT and SIFT-OCT are used to extract CPs in the master image (‘C’) and slave image (‘G’). However, the spatial distributions of the points from the SIFT and SIFT-OCT are quite similar, and presenting both results is not necessary. To clarify the proposed matching scheme is promising. The results of the original SIFT, which has been proven unsuitable for SAR, are presented and compared with the one-step scheme. The results of the different algorithms for the traditional one-step matching and proposed two-step matching are presented in Table 1. It can be seen from Table 1 that even for the original SIFT algorithm, which has been proven unsuitable for SAR image registration, the registration accuracy was substantially improved after the two-step matching method proposed in this paper, which is not much different from the registration accuracies of the other algorithms specifically proposed for SAR image registration. Thus, without specific instruction, only the results of the SIFT are presented in the following figures. The extracted keypoints of the SIFT are shown in Figure 4. In the master image, 25,096 and 7303 keypoints from the SIFT and SIFT-OCT, respectively, were extracted. In the slave image, 21,294 and 6589 keypoints from the SIFT and SIFT-OCT, respectively, were extracted. As the SIFT-OCT skipped the first octave of a scale-space pyramid, the number of keypoints from the SIFT-OCT is much smaller than that of the SIFT. However, the keypoints from the SIFT-OCT are more robust to multiplicative speckle noise than those of the SIFT.

**Figure 5:**
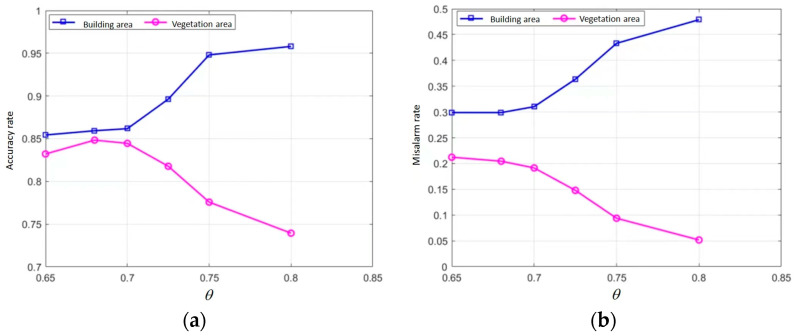
Relation between weights and matching results. (**a**) The relation of threshold and accuracy of matching points; (**b**)The relationship between the threshold and false reporting rate.

**Figure 6:**
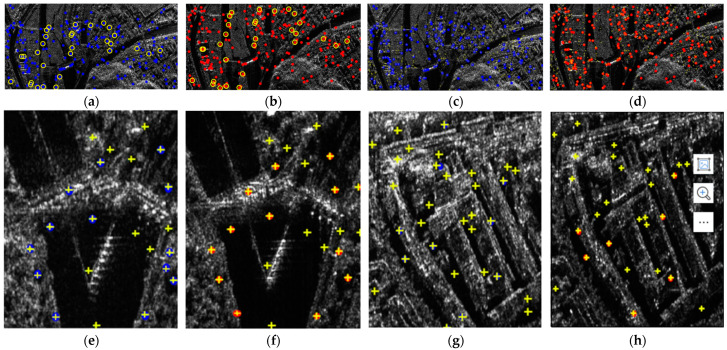
CP matching comparisons of SIFT. (**a**,**b**) are global-matching results, where the blue and red points are dual-matched CPs, and the yellow-circled points are dual-matched points filtered by RANSAC. (**c**,**d**) are local-matching and global-matching results, respectively, and blue and red points are the same as in (**a**,**b**); the yellow crosses are local-matching results. (**e**–**h**) are some details of (**c**,**d**).

The value of the threshold (θ) may affect the final registration results; thus, this paper examines the effect of the θ on the matching results. The relationship between the θ and the accuracy of the matching points is shown in Figure 5a, and the relationship between the θ and false-alarm rate is shown in Figure 5b. With the increase in the θ, the accuracy of the matching points of the matching rate of vegetation decreased. To achieve the matching accuracy of both buildings and vegetation, the value of the θ was selected as 0.7. In the global dual-matching procedure, the threshold (θ) was set to 0.7, and 218 and 204 pairs of CPs from the SIFT and SIFT-OCT, respectively, were selected. The SIFT results are shown in Figure 6a,b with the blue and red points, respectively. The global dual-matching cost is 273.35 s for the SIFT and 28.82 s for the SIFT-OCT. After the RANSAC step, only 21 CPs for the SIFT and 26 CPs for the SIFT-OCT were selected. The yellow circled points in Figure 6a,b are the selected global CPs of the SIFT. They cost 6.59 s and 3.71 s, respectively. From Figure 6a,b, we can see that some of the global dual-matched CPs were mismatched. The mismatched CPs were filtered by the RANSAC procedure. However, some correct CPs were also filtered. The global affine transformation parameters were estimated by the selected 21 or 26 CPs. By transforming the keypoint location of the slave image into the master image coordinate system, the location information can be used to confine the searching scope. In the local-matching procedure, CPs of the other image within a radius of 100 pixels are searched, and the threshold (θ) of the local dual match is also set to 0.7. After the local-matching procedure, 437 and 457 CPs for the SIFT and SIFT-OCT were selected, respectively (see the yellow crosses in Figure 6c,d). Compared with the global-matching processing times (273.35 s and 28.82 s, respectively), the local-matching processing times were only 74.35 s and 5.15 s, respectively. As many similar CPs outside of the search area are already eliminated, the local-matched CPs are more robust than the global dual-matched CPs. Details of Figure 6c,d can be seen in Figure 6e–h. An interesting phenomenon appears in both the SIFT and SIFT-OCT methods: all the points selected by global matching after the RANSAC (21 for the SIFT and 26 for the SIFT-OCT) are also selected by local matching. The local matching also kept many other high-quality CPs, which could not be matched by global matching. The larger number of CPs (i.e., 437 − 21 = 416 and 457 − 26 = 431 for the SIFT and SIFT-OCT, respectively), which could solve the problem of complex distortion, is a significant improvement compared with one-step matching.

**Figure 7:**
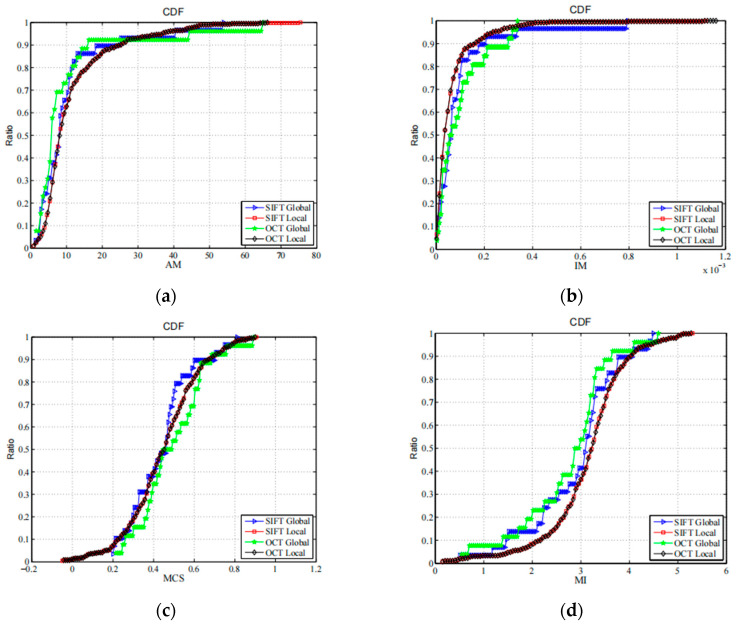
CP CDFs for different similarity measurement functions for all four measurements; higher values correspond to more similar patches: (**a**) AM; (**b**) IM; (**c**) MCS; (**d**) MI.

The normal method to evaluate the quality of matched CP pairs is to use match error, (i.e., root mean square error). However, the correct tie point, which cannot be determined in VHR images, even by manual selection, must be known. Thus, other methods, such as similarity measurements, are alternatively used to perform the evaluation. The high similarity of the extractor features are ensured in the matching scheme. Thus, some other similarity measurement functions are used to evaluate the performance of the CPs: the alignment metric (AM) [59], invariant moment (IM) [60], matching correlation surface (MCS) [61], and mutual information (MI) [62]. For these measurements, higher values correspond to the higher similarity of two image patches. For each CP, a pair of self-centered image patches are selected to calculate the similarities, and in these experiments, the patch size is 31 × 31. The cumulative distribution functions (CDFs) of these four similarities from both the SIFT and SIFT-OCT are shown in Figure 7, and some of their corresponding statistical parameters, including maximums, minimums, and means, are presented in Table 2. Even though the numbers of CPs of the different methods are not the same, CDF curves can show the similarity distribution of extracted CPs, which can be used to evaluate their overall quality from one method. In Figure 7, we can see that the curves of the local matching from the SIFT and SIFT-OCT are quite close, which reflect that the local matching of both the SIFT and SIFT-OCT had similar performances. In Figure 7a,d, the local curves of the AM and MI are almost below the global curves, which reflect the better similarity of the local-matched CPs. In Figure 7c, two local curves are in the middle of two global curves, which means that the local-matched CPs have moderate similarity measured by the MCS. The local curves in Figure 7b are slightly above both global curves, which should mean that the global-matched CPs have better similarities measured by the IM. However, in Figure 7b, all four of these curves are very steep in small values, which means that the differences in these four curves are very small. We can conclude that the similarity of the local-matched CPs is not lower than that of the global-matched CPs. Thus, compared with traditional one-step matching, the proposed two-step matching increases the number of CPs while improving the matching accuracy.

**Figure 8:**
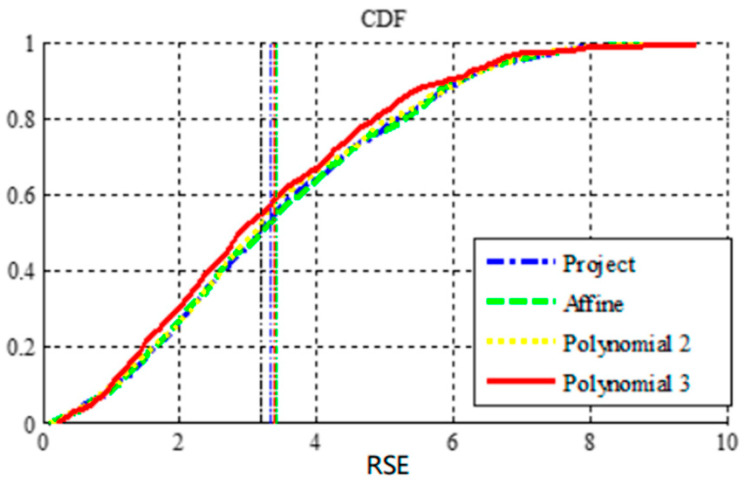
Cumulative distribution curve of mean partition error of control points after registration of global function.

To show that the simple model cannot describe complex geometric distortion, the SIFT algorithm locally matched the 437 control points, and the least-squares method was used to estimate the parameters of multiple global transformation models. The probability accumulation function (CDF) of the root square error (RSE) of the control point after registration was calculated. The experimental results are shown in Figure 8. It can be seen from the figure that the best registration accuracy can be achieved by using the third-order polynomial; however, the mean square errors of most control points are still very high. Despite the large number of control points used in the experiment, the global-distortion model still did not achieve high registration accuracy.

**Figure 9:**
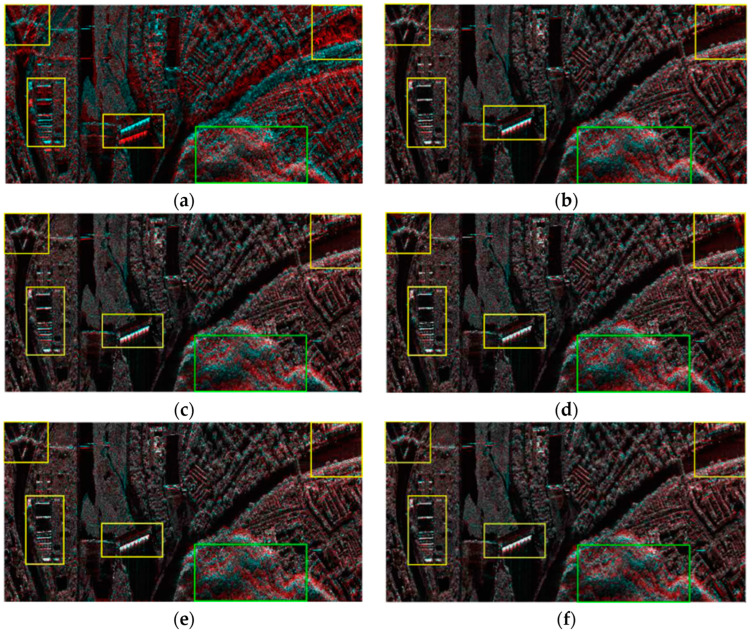
Registration results compared with pseudoimages of main image and auxiliary image. The main image is the red channel, and the auxiliary image is the blue and green channels. (**a**) Original unregistered image. (**b**) Auxiliary image obtained through global affine transformation registration. Affine transformation parameters were estimated from 21 control points obtained by global matching of the SIFT algorithm. (**c**) Auxiliary image obtained through global affine transformation registration. Affine transformation parameters were estimated from 437 control points obtained by local matching of the SIFT algorithm. (**d**) Auxiliary images registered by triangle network and affine transformation. (**e**) Auxiliary images by TPS registration. (**f**) Auxiliary images by local affine transformation registration.

**Figure 10:**
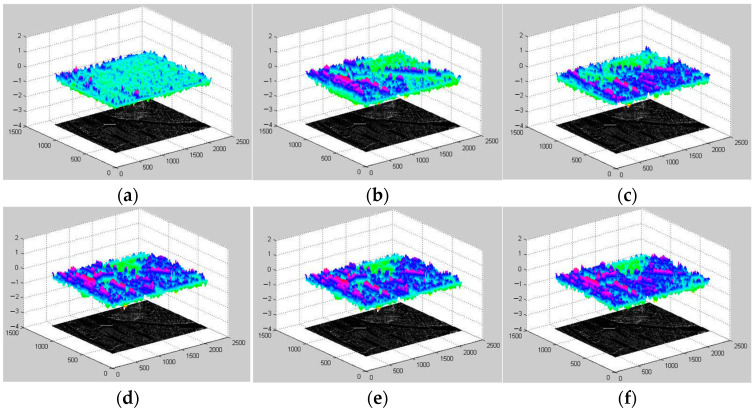
Comparison of registration correlation coefficients. (**a**) Raw unregistered images. (**b**) Auxiliary images obtained through global affine transformation registration. Affine transformation parameters were estimated from 21 control points obtained by global matching of the SIFT algorithm. (**c**) Auxiliary image obtained by global affine transformation registration. Affine transformation parameters were estimated by 437 control points obtained by local matching of the SIFT algorithm. (**d**) Auxiliary image obtained by triangle network and affine transformation registration. (**e**) Auxiliary image obtained by TPS registration. (**f**) Auxiliary image obtained by local affine transformation registration.

Due to the inaccessible point-to-point correspondence between the two images in the real case, two methods were used to evaluate the registration accuracy in the experiments presented in this paper. One uses the pseudocolor method to synthesize the main image and registered auxiliary image into pseudocolor to allow for direct analysis with the human eye. The other calculates the correlation coefficient of each point in the image; the higher the absolute correlation coefficient, the higher the registration accuracy. The comparative results of this experiment are shown in Figure 9 and Figure 10. Figure 9a and Figure 10a show the evaluation results of the original data. It can be seen that the main images and auxiliary images are completely unaligned, there are many overlapping images, and the correlation coefficient is small. Figure 9b,c and Figure 10b,c are the coarse registration results. It can be seen that the two images basically match; however, there are still some overlapping images in some strong scattering regions, and the overall correlation coefficient is not high. Because both used global affine transformation as the distortion model, although the number of control points in the two groups varies greatly, the results are not different. In the case of the distortion model, the accuracy is not high, even if a large number of control points are used, and the overall registration accuracy cannot be improved. The registration results of the model parameter estimation using triangular net, spline interpolation, and local affine transformations as models, and 437 control points obtained using SIFT, are shown in Figure 9d–f and Figure 10d–f. It appears from the figure that the registration results obtained by using the local-distortion model are better than those obtained with the global-distortion model. In the yellow-box areas, the local affine model and spline value are better than the affine transformation. Because no control points are found in the image boundary region, the triangle net affine transformation results are poor in this region. The local affine transformation model alleviates this problem by increasing the number of control points slightly farther away. In the areas marked by the green boxes, all the methods have poor results, which is mainly because the area is mountainous with a large number of trees, and the SAR images, taken from different angles, are different without a substantial difference in the large number of control points obtained. A quantitative analysis of the results is presented in Table 3.

**Figure 11:**
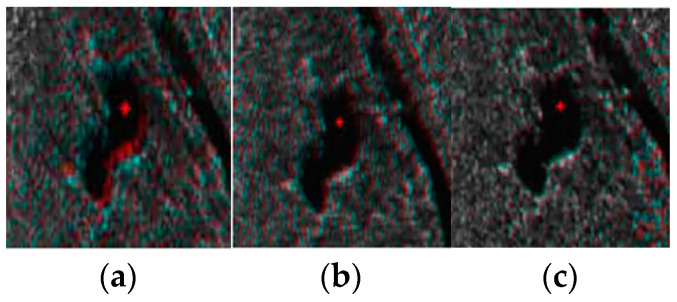

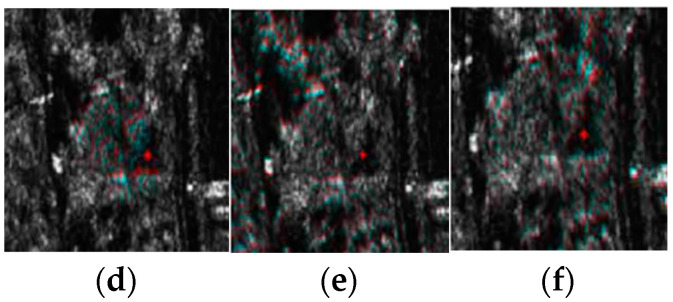
Comparison of robustness of different models after increasing offset of control points: (**a**) result of using triangle net affine transformation after increasing error in Region 1; (**b**) result of using TPS interpolation after increasing error in Region 1; (**c**) result of using local affine transformation after increasing error in Region 1; (**d**) result of using triangle net affine transformation after increasing error in Region 2; (**e**) result of using TPS interpolation after increasing error in Region 2; (**f**) result of using local affine transformation after increasing error in Region 2.

To compare the robustness of the three local distortion models to the mismatching points, three control points were added for the pixel offset of the mismatching in the image. The local results after matching are shown in Figure 11. From the results, we can see that the local affine model has a higher stability relative to the triangular net affine transformation and spline interpolation. The reason is that both spline interpolation and the triangle network transform the coordinates of all the control points to consistency, and if mismatched, the control points can distort the image of that region. However, the local affine transformation model uses more control points to estimate the transformation parameters, and it has a certain tolerance to the matching error. A quantitative analysis of the results is presented in Table 4.

### 3.3. Registration Result

**Figure 12:**
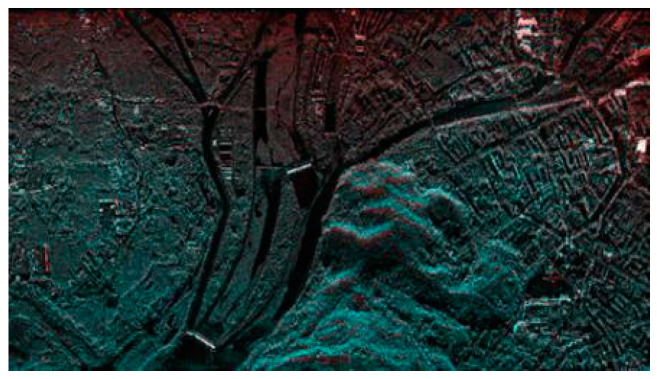
Registration result of dataset using SIFT and two-step matching.

Using TPS [30] as a model, the experiment images were registered by cubic interpolation. The parameters of the model were estimated by CPs extracted from our proposed two-step matching scheme. The registration result of the experiment dataset is shown in Figure 12. The figure only retained the overlapping areas by putting the registered master image in the red channel and the slave image in the cyan channel. If the two images are exactly the same, then the overlapped synthetic image should be a grayscale image. Moreover, if two overlapped images are displaced, the synthetic image should have clear red and cyan shadow pairs. Thus, this synthetic image is suitable for the evaluation of the registration result. In Figure 12, the synthetic image has almost no shadow pairs, which means that the two images are precisely registered. In addition, the grayscale images are not identical, which indicates that the intensities of the two image areas are quite different in various locations. The grayscale levels of the different range gates from the same image are not identical because the antenna pattern weight was not entirely corrected (which caused the top of Figure 12), has some stronger red component, and the bottom has stronger cyan component. The experiment demonstrates that, with a larger quantity of good-quality CPs from SAR images suffering from geometrical deformation and intensity differences, the proposed method can obtain high-precision registration results.

**Figure 13:**
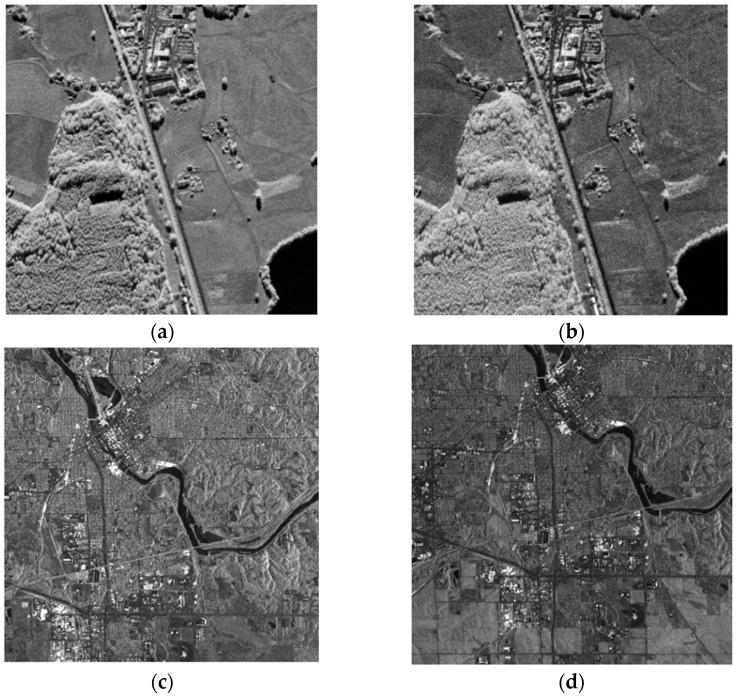
SAR images used for experiments: (**a**) master image for first set of data; (**b**) slave image for first set of data; (**c**) master image for second set of data; (**d**) slave image for second set of data.

In order to verify the registration performance of the proposed algorithm, two excellent methods in SAR image registration were selected as comparative experimental methods. SAR-SIFT [24] is the method that has been used to achieve good SAR image registration performances in recent years. It is proposed in the framework of SIFT, and it adopts the mean proportion operator to overcome the spot noise to extract multiscale stable angle points. However, BFSIFT [22] extracts local extreme points in the nonlinear scale space for SAR image registration. The experimental data were registered by different registration methods to verify the superiority of the proposed registration method. The first set of data is the airborne SAR images from the DLR Microwave Radar Research Institute. Images were taken near a small city, in southern Bavaria, Germany, and they contain different objects, such as forests, farmland, water bodies, and houses. Data were recorded from two images at different times, in different bands, and in different polarization situations, and the resulting images are significantly different, as shown in Figure 13. Figure 13a is the reference image, and Figure 13b is the image to be registered. The reference image and registered image of the second set of data were taken in June 2008 and July 2008, respectively, in Iowa, the United States. The reference map and registered map are shown in Figure 13c,d, respectively.

**Figure 14:**
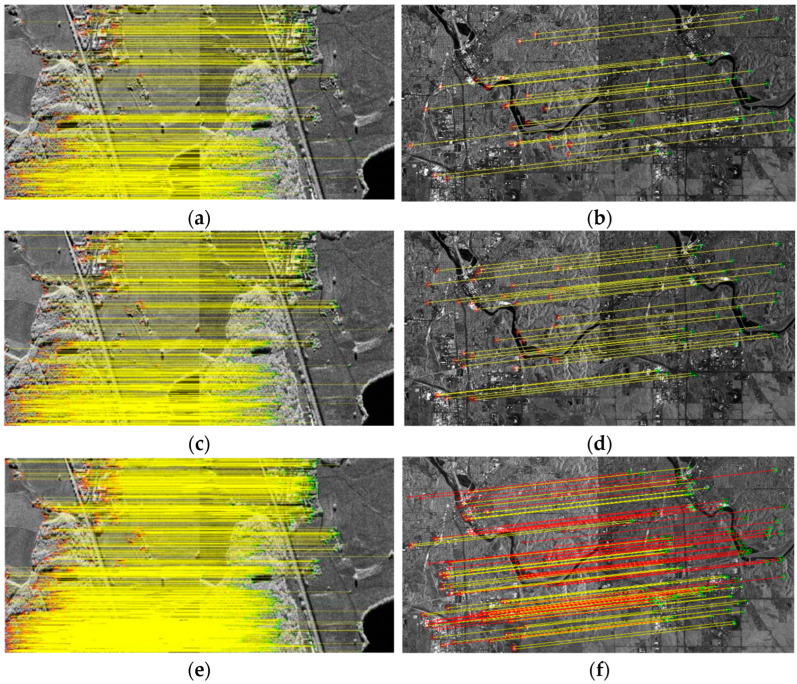
Matching points obtained from two sets of experimental data: (**a**) obtained matching point pairs by the BFSIFT algorithm for the first set of data; (**b**) obtained matching point pairs by the BFSIFT algorithm for the second set of data; (**c**) obtained matching point pairs by the SAR-SIFT algorithm for the first set of data; (**d**) obtained matching point pairs by the SAR-SIFT algorithm for the second set of data; (**e**) obtained matching point pairs by proposed method for the first set of data; (**f**) obtained matching point pairs by proposed method for the second set of data.

The matching points obtained for two sets of experimental data using BFSIFT, SAR-SIFT, and the method presented here are shown in Figure 14. The matching point pairs obtained with the BFSIFT and SAR-SIFT methods are shown in Figure 14a–d, respectively. The red circle is the matching point in the reference image, the green cross is the matching point in the graph to be registered, and the yellow line connects the reference map with the matching point pair in the graph to be registered. Figure 14e,f shows the matching point pairs obtained by the proposed method. The yellow line in Figure 14f is the line with more feature points than Figure 14b,d, and the red line is the line with the same number of feature points as Figure 14b,d. It can be seen from the figure that the method in this paper can identify more pairs of matching points, making the distribution of matching points more uniform.

**Figure 15:**
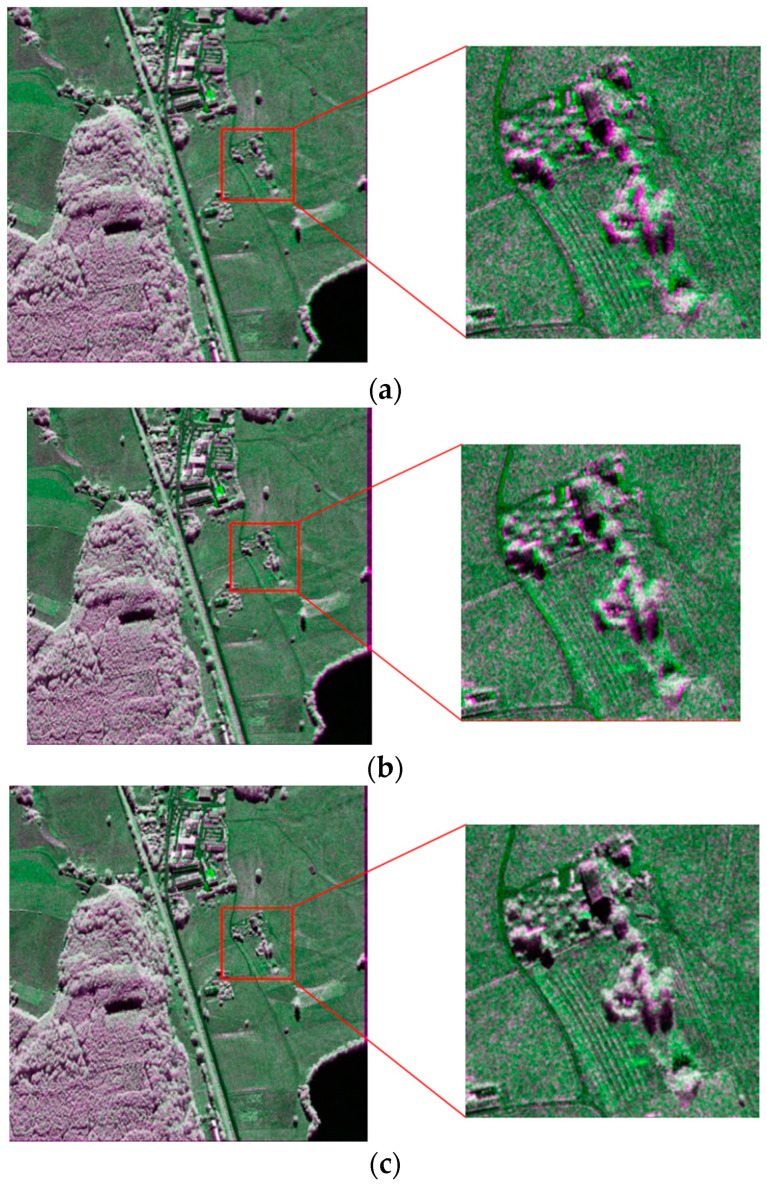
First set of data registration results: (**a**) BFSIFT registration results; (**b**) SAR-SIFT registration results; (**c**) proposed-method registration results.

**Figure 16:**
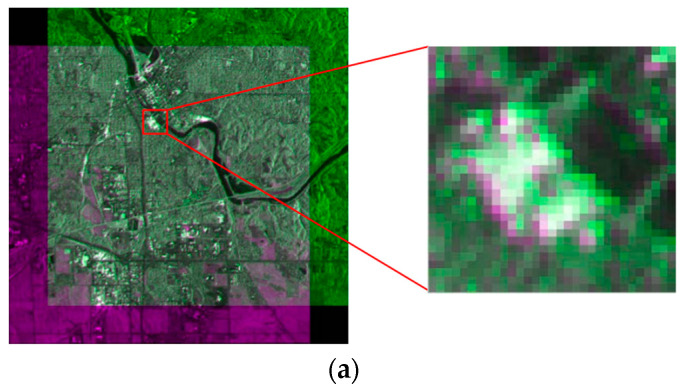

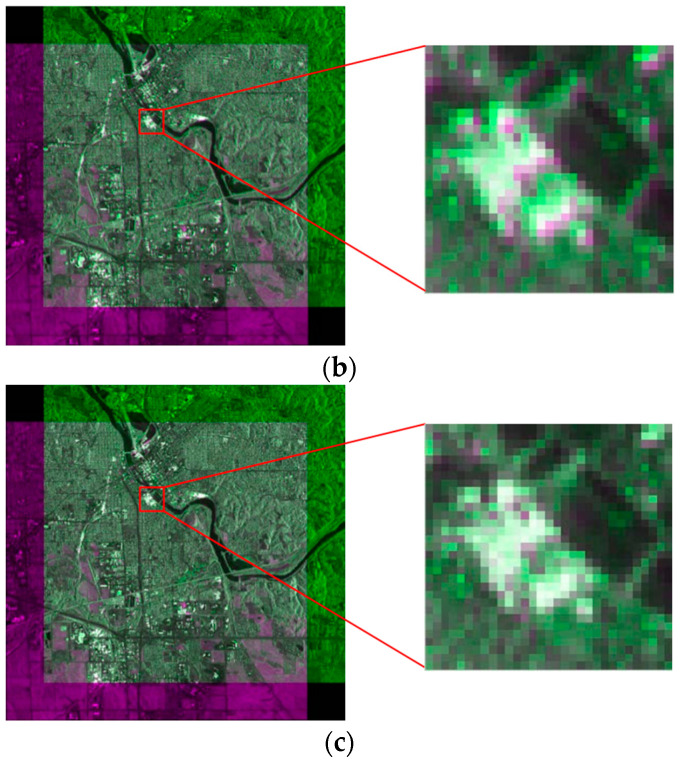
Second set of data registration results: (**a**) BFSIFT registration results; (**b**) SAR-SIFT registration results; (**c**) proposed-method registration results.

Figure 15 and Figure 16 show the resulting pseudocolor plots of the registration to the two sets of experimental data using BFSIFT, SAR-SIFT, and the method proposed here. In the pseudocolor diagram, green indicates the reference image, and purple represents the image to be registered. The two images are overlapped so that the registration effect of each method can be observed more clearly. The red rectangle box in the left image is the position of the right zoom-in in the original image. The SAR image registration obtained by the BFSIFT method and SAR-SIFT method did not accurately align the image to be registered to the reference image. The two overlapping images on the pseudocolor map have an obvious alignment error. The registration results of the proposed method have the best registration effect, and the reference image overlaps the image to be registered well.

As can be seen from Table 5, in two sets of experiments, BFSIFT and SAR-SIFT can only extract a single feature for registration, the number of matching feature points is small, and the insufficient number of feature points are not effectively evenly distributed in the image, which affects the accuracy of the final registration results. The matching method proposed here adopts a two-step matching strategy under a SIFT-like SAR image registration scheme to obtain more control points and thus obtain accurate registration results. It also can be seen from the registration result graph and registration data table that the registration algorithm proposed in this paper is better than the BFSIFT and SAR-SIFT methods under equivalent conditions.

**Figure 17:**
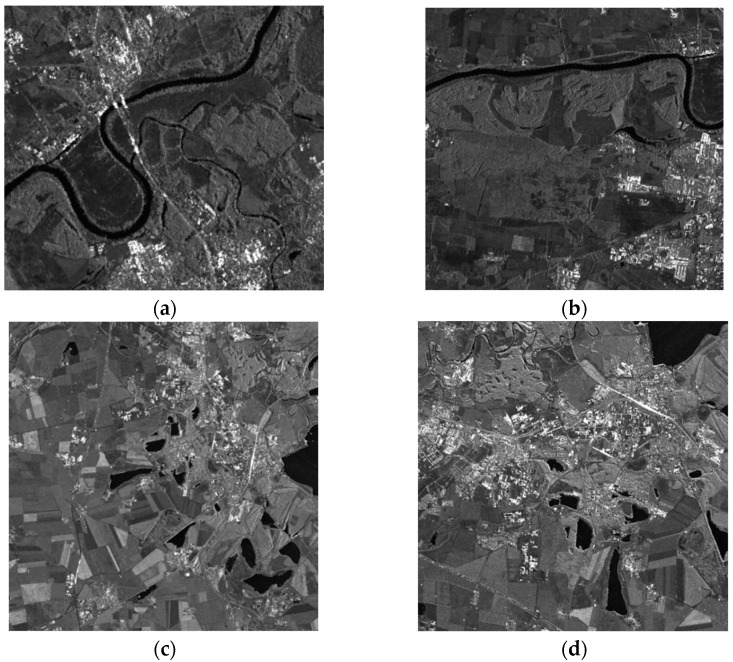
SAR images used for experiments: (**a**) master image for first set of data; (**b**) slave image for first set of data; (**c**) master image for second set of data; (**d**) slave image for second set of data.

Through the above experiment, we can see that the two-step matching strategy proposed in this paper can obtain a large number of control points and, at the same time, greatly improve the image matching accuracy; however, the increase in the number of CPs must lead to the subsequent feature calculation and matching calculation. Therefore, in the two-step-matching strategy framework, the RANSAC [34] method is added to remove false matching, eliminate some of the wrong matching points, and improve the calculation rate. To verify that the proposed algorithm does not increase the computation too much while improving the matching accuracy, and that is has a good registration performance, two sets of images were selected for comparison experiments. The first set of data is the airborne SAR image of a certain area of Serbia. In the image are a plain area and river. There is a large translation transformation and a certain amount of rotation transformation and scale transformation between the two images, and the overlap between them is small. The reference image and image to be registered are shown in Figure 17a,b, respectively. The second set of data is the airborne SAR image of a certain area in Germany, which contains substantial farmland and a lake in the plain area. The overlap between the two images is relatively large. The reference image and image to be registered are shown in Figure 17c,d, respectively.

**Figure 18:**
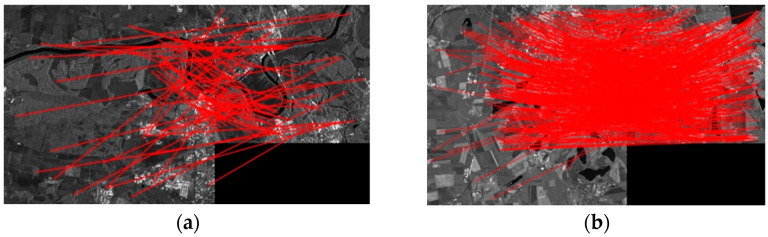

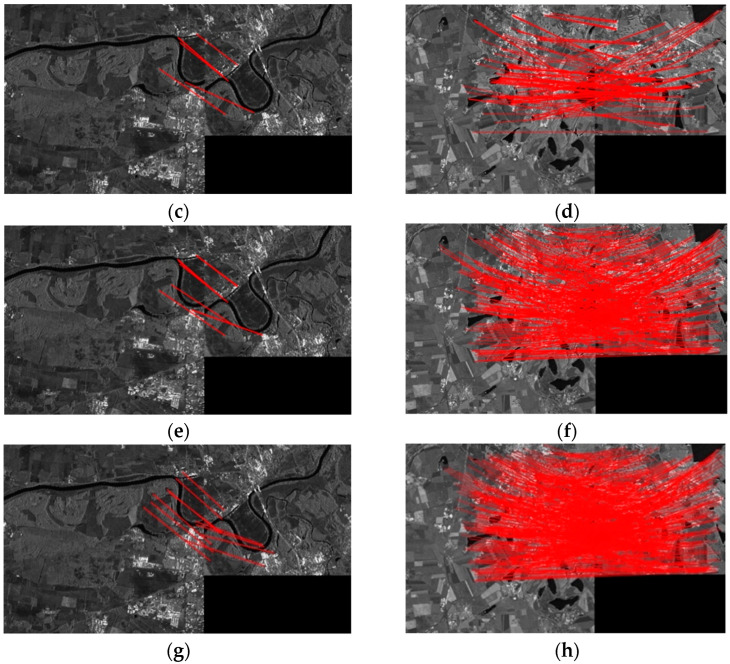
Matching points obtained from two sets of experimental data: (**a**) obtained matching point pairs by the SIFT algorithm for the first set of data; (**b**) obtained matching point pairs by the SIFT algorithm for the second set of data; (**c**) obtained matching point pairs by the SIFT-OCT algorithm for the first set of data; (**d**) obtained matching point pairs by the SIFT-OCT algorithm for the second set of data; (**e**) obtained matching point pairs by the SAR-SIFT algorithm for the first set of data; (**f**) obtained matching point pairs by the SAR-SIFT algorithm for the second set of data; (**g**) obtained matching point pairs by the proposed method for the first set of data; (**h**) obtained matching point pairs by the proposed method for the second set of data.

The matching points obtained for two sets of experimental data using SIFT, SIFT-OCT, SAR-SIFT, and the method presented here are shown in Figure 18. In Table 6, the SIFT algorithm, SIFT-OCT algorithm, SAR-SIFT algorithm, and proposed algorithm were applied for two sets of images to compare the matching effects of the SAR image pairs in the experimental data, and the evaluation indexes of the matching effects were calculated.

**Figure 19:**
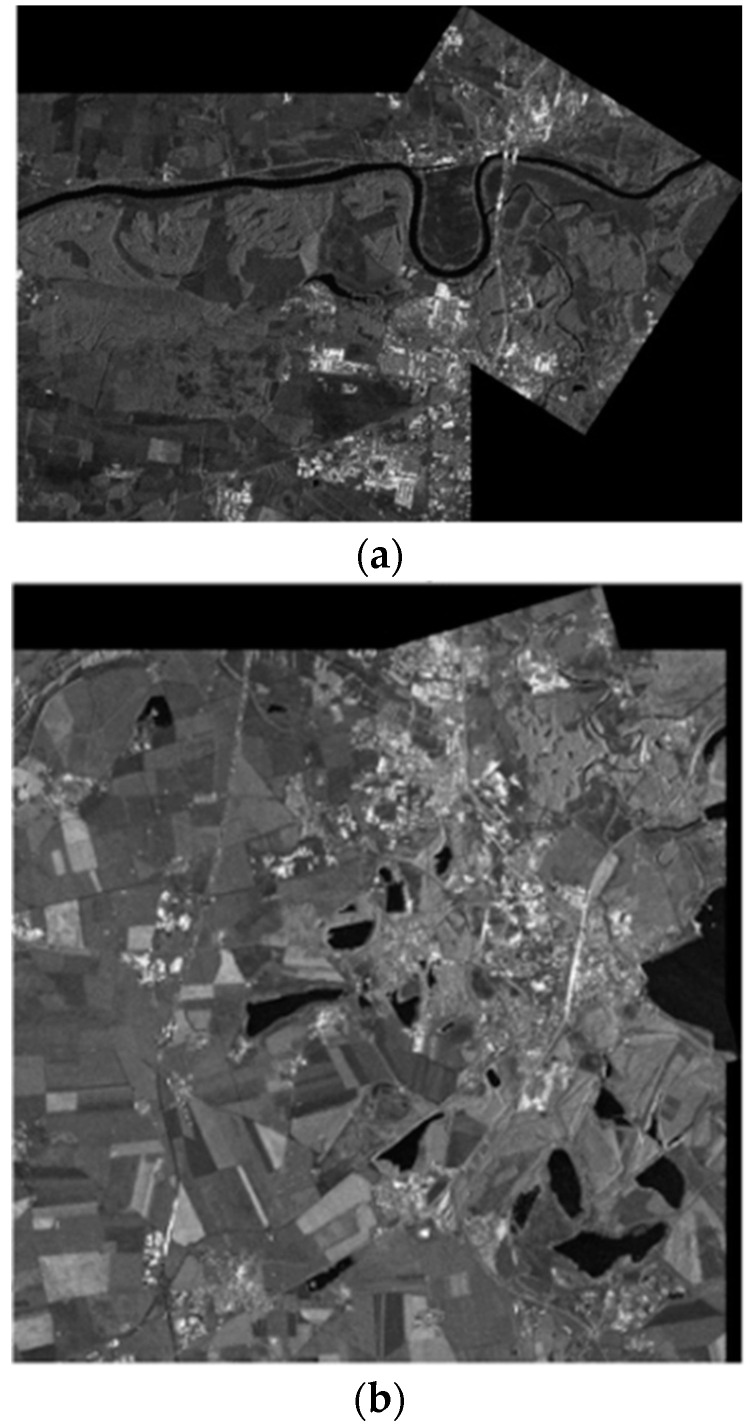
Contour overlays of registration results of two pairs of images using the proposed algorithms. (**a**) The first set of data registration results; (**b**) The second set of data registration results.

Judging from the results of the matching index calculation, the proposed two-step matching algorithm increases the number of correct matching points. Because it extracts a large number of feature points for matching and introduces the RANSAC method to remove false matching, it also saves the running time of matching under the premise of ensuring the correct matching rate. Using the classic SIFT algorithm to match the SAR images, a large number of wrong matching pairs appeared, which finally caused registration failure. When the image has fewer overlapping areas and matching points, the registration accuracy of the SIFT-OCT algorithm is higher than that of the SAR-SIFT algorithm. This is because the SAR-SIFT algorithm retains a large number of wrong matching pairs, which results in a decrease in the matching accuracy. When the overlap of the registration image is large, the matching accuracy of the SAR-SIFT algorithm is greatly improved. The two-step matching algorithm proposed in this paper has higher matching accuracy than those of several other algorithms in these two sets of SAR image matching, and there is little difference in the matching time. The matching results of the two sets of images are shown in Figure 19.

**Figure 20:**
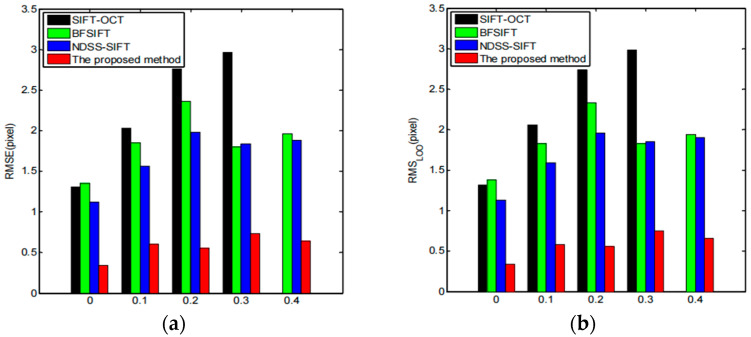
Comparison of registration results at different noise levels: (**a**) RMSE; (**b**) RMS_LOO_.

**Figure 21:**
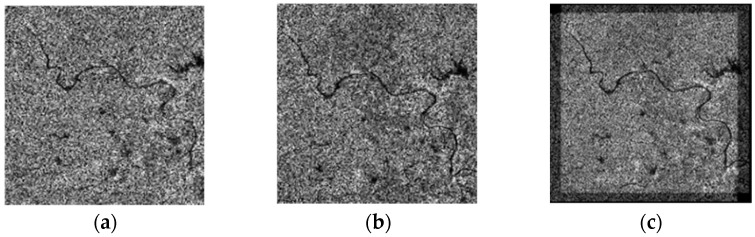
Image registration results with noise variance of 0.4: (**a**) reference image; (**b**) image to be registered; (**c**) registration result.

Next, we tested the robustness of the registration method proposed here to high-speckle noise. For the test purpose, spot noise with different noise variances was manually added to a set of multitemporal-phase-measured SAR images, and the noise variance was set to 0, 0.1, 0.2, 0.3, and 0.4. The test images used were acquired from the C-band images by the US AIRSAR system. Four SAR image registration methods were used: the SIFT-OCT method, BFSIFT method, NDSS-SIFT method, and proposed registration method, for the registration of the above four sets with different noise levels of SAR images. The results of the obtained registration evaluation are shown in Figure 20. According to the performance evaluation results, we can see that the SIFT-OCT method registration failed when the noise variance was greater than 0.3, but the proposed registration method still maintained a good registration performance. It can be seen from the values of the RMSE and RMS_LOO_ that the registration accuracy proposed here is much higher than those of the SIFT-OCT method, BFSIFT method, and NDSS-SIFT method. Figure 21 shows the registration results of the image when the noise variance was equal to 0.4. The obtained registration results basically match the edges and textures in the reference images, which also indicates that the registration method in this chapter is robust to spot noise.

## 4. Conclusions

The difficulty of SAR image registration increases with an increase in the resolution. In recent years, a large number of star-borne and airborne SAR systems have emerged, and the observation of SAR images has become clearer. The improvement in the resolution makes the image matching more difficult. According to the difficulty of airborne high-resolution multiphase SAR image registration, the distortion model is decomposed into global distortion and local distortion. The SIFT family feature extraction method is used to obtain feature points, and the causes of interference under control point matching are analyzed. A two-step matching algorithm combining global matching and local matching is proposed to obtain a large number of matching control points. The quality of the control points obtained by global matching and local matching, and the accuracies of different distortion models, were evaluated to verify the effectiveness of the proposed method.

In this paper, we focus on increasing the quantity of CPs for VHR SAR image registration, which suffers from complex distortion. Instead of improving the feature extraction method, this study paid more attention to the keypoint matching scheme, which can also improve the CP detection performance. The proposed two-step matching scheme was tested on SIFT and SIFT-OCT. Compared with traditional one-step global matching, our proposed two-step matching method can obtain a significantly larger number of high-quality CPs, which are evaluated by four similarity measurement functions. C-band 0.5 m resolution Chinese airborne SAR images with different acquisition times and imaging geometries are precisely registered by our proposed method. The large number of control points obtained using the two-step matching method is comparable to the number of global-matching control points. An analysis of the distortion model shows that using a simple global distortion model does not simulate a complex deformation. However, a local affine transformation enhances the robustness to noise with constant accuracy, which is more suitable for complex registration application scenarios. By using a large quantity of good-quality CPs, SAR images suffering from geometrical deformation and intensity differences can also obtain high-precision registration results.

## Figures and Tables

**Table 1:** Results of different algorithms for traditional one-step matching and proposed two-step matching.

Algorithm	One Step			Two Steps		
	Matching Points	Correct Points	Correct Rate (%)	Matching Points	Correct Points	Correct Rate (%)
SIFT	21	9	42.9	437	388	88.8
SIFT-OCT	26	22	84.6	457	432	94.5
SAR-SIFT	192	159	82.8	4013	3809	94.9
PCA-SIFT	51	35	68.6	989	891	90.1
BF-SIFT	137	110	80.3	2815	2640	93.8

**Table 2:** CP similarity measurement comparison (G: global matching; L: local matching).

		AM		IM (10^−5^)		MCS (10^−1^)		MI	
		G	L	G	L	G	L	G	L
Max	SIFT	53.9	75.4	79.5	111.7	8.1	9.1	4.5	5.3
	SIFT-OCT	65.0	66.1	33.7	116.1	8.9	9.0	4.6	5.3
Min	SIFT	1.3	0.8	0.3	0.3	2.0	0.5	0.5	0.2
	SIFT-OCT	1.5	0.8	0.3	0.1	2.1	0.4	0.5	0.2
Mean	SIFT	10.9	12.0	10.1	7.0	4.4	4.5	2.9	3.2
	SIFT-OCT	10.4	11.0	10.0	7.4	4.9	4.4	2.7	3.1

**Table 3:** Quantitative analysis of results (G: global matching; L: local matching).

Method	Matching Points	Correct Points	Correct Rate (%)
SIFT ^G^	21	15	71.4
SIFT ^L^	437	338	72.8
SIFT ^L^ (triangle network)	437	372	85.1
SIFT ^L^ (TPS)	437	395	90.4
SIFT ^L^ (proposed)	437	407	93.1

**Table 4:** Quantitative analysis of results.

Region	Method	Matching Points	Correct Points	Correct Rate (%)
Region 1	Triangle network	23	19	82.6
	TPS	23	20	87.0
	Proposed	23	22	95.7
Region 2	triangle network	27	23	85.2
	TPS	27	24	88.9
	proposed	27	25	92.6

**Table 5:** SAR image registration data.

Method	First Set of SAR Images		Second Set of SAR Images	
	Matching Points	RMSE	Matching Points	RMSE
BFSIFT	437	1.28	27	1.19
SAR-SIFT	573	1.13	34	0.93
Proposed	947	0.65	97	0.47

**Table 6:** Comparison of matching effects of the SIFT, SIFT-OCT, SAR-SIFT, and proposed algorithms on two image pairs. * represents failure to match.

			SIFT	SIFT-OCT	SAR-SIFT	Proposed
First set of data	CPs	Master image	214	267	349	503
		Slave image	165	196	247	315
	Matching pairs		81	20	40	32
	Correct matching pairs		*	6	9	11
	Match time/s		1.3425	1.683	1.945	2.094
	CMR		*	0.300	0.225	0.344
Second set of data	CPs	Master image	1053	905	1204	1865
		Slave image	907	490	661	1002
	Matching pairs		809	253	401	318
	Correct matching pairs		*	134	296	248
	Match time/s		6.6936	5.802	7.099	8.2734
	CMR		*	0.530	0.738	0.780

## Data Availability

Not applicable.
